# Implementing chlamydia screening: what do women think? A systematic review of the literature

**DOI:** 10.1186/1471-2458-6-221

**Published:** 2006-09-01

**Authors:** Natasha L Pavlin, Jane M Gunn, Rhian Parker, Christopher K Fairley, Jane Hocking

**Affiliations:** 1 Department of General Practice, Melbourne University, 200 Berkeley St, Carlton, Melbourne, Australia; 2 School of Population Health, Melbourne University, Carlton, Melbourne, Australia; 3 Melbourne Sexual Health Centre (MSHC), 580 Swanston St, Carlton, Melbourne, Australia; 4 Centre for Epidemiology and Population Health Research, Burnet Institute, 85 Commercial Road, Melbourne, Victoria 3001, Australia

## Abstract

**Background:**

*Chlamydia trachomatis *is a common sexually transmitted infection that can have serious consequences. It is universally agreed that screening for chlamydia infection should be offered to sexually active young women. We undertook a literature review to document the views, attitudes and opinions of women about being screened, tested and diagnosed with *Chlamydia trachomatis*.

**Methods:**

Online databases (MEDLINE, Meditext, PsycINFO, Web of Science) and reference lists searched up to August 2005. Search terms: chlamydia, attitude, attitude to health, interview, qualitative, women. Eligibility criteria: about chlamydia, included women, involved interviews/surveys/focus groups, looked at women's views/opinions/attitudes, published in English. Thematic analysis identified the main and recurrent themes emerging from the literature. We compared our thematic analysis with the Theory of Planned Behaviour to provide a model that could assist in planning chlamydia screening programs.

**Results:**

From 561 identified articles, 25 fulfilled inclusion criteria and were reviewed. 22: USA, UK; 3: Holland, Sweden, Australia. Major themes identified: need for knowledge and information, choice and support; concerns about confidentiality, cost, fear, anxiety and stigma. Women are more likely to find chlamydia screening/testing acceptable if they think chlamydia is a serious, common condition which can cause infertility and if they understand that chlamydia infection can be asymptomatic. Women want a range of options for chlamydia testing including urine tests, self-administered swabs, pelvic exams and clinician-collected swabs, home-testing and community-based testing. Tests should be free, easy and quick. Women want support for dealing with the implications of a chlamydia diagnosis, they feel chlamydia diagnoses need to be normalised and destigmatised and they want assistance with partner notification. Women need to know that their confidentiality will be maintained.

**Conclusion:**

Our review found that women from various countries and ethnic backgrounds share similar views regarding chlamydia screening, testing and diagnosis. The acknowledged importance of women's views in planning an effective chlamydia screening program is expanded in this review which details the nature and complexity of such views and considers their likely impact.

## Background

*Chlamydia trachomatis *is a common sexually transmitted infection that can have serious consequences [1]. The advent of nucleic acid amplification technology has made rapid, sensitive, non-invasive testing available. Effective treatment is also available and is safe and simple to use [2]. Chlamydia satisfies the WHO criteria for screening and many countries around the world already have screening programs in place, including Sweden [3], USA [4,5] and UK[6]. Many others including the Netherlands [7], Denmark [7] and Australia are still considering how best to implement chlamydia screening. Chlamydia is a significant health problem in Australia. Notification rates have increased 4 fold between 1997 and 2005 from 47.4 per 100,000 to 203 per 100,000 in 2005 (40,917 notifications) [8]. Australian data show that the greatest burden of chlamydia infection is among young women aged 16 to 24 years, with over two thirds of chlamydia notifications among women in 2005 being in this age group [8]. Australian prevalence surveys have also shown that chlamydia prevalence is higher among young women [9].

Even in countries such as Sweden where chlamydia screening has been established since the 1980s, uptake varies [10]. Successful programs require education of both health professionals and the community and ultimately need to be informed by the views of the groups being screened. It is universally agreed that sexually active young women (up to at least the age of 25) are an important target group. Although there are numerous published studies looking at various aspects of women's views on chlamydia screening, to date there are no published reviews available.

Understanding why people do or do not take up chlamydia screening can be assisted using existing psychological theory such as the Theory of Planned Behaviour (TPB) originally proposed by Ajzen [11]. TPB suggests that an individual's behaviour is due to Attitude: whether the person is in favour of the behaviour; Subjective Norms: whether the person perceives that society is in favour of the behaviour, and to Perceived Behavioural Control: the extent to which the person feels able to enact the behaviour.

Our aim was to summarise and qualitatively analyse the published international literature examining the views, attitudes and opinions of women screened, tested and diagnosed with *Chlamydia trachomatis*. We aimed to build an evidence base to inform the design of an appropriate and effective chlamydia screening program in Australia [12]. We used the TPB to synthesise our findings into a model to assist in planning chlamydia screening programs [11].

## Methods

**Figure 1:**
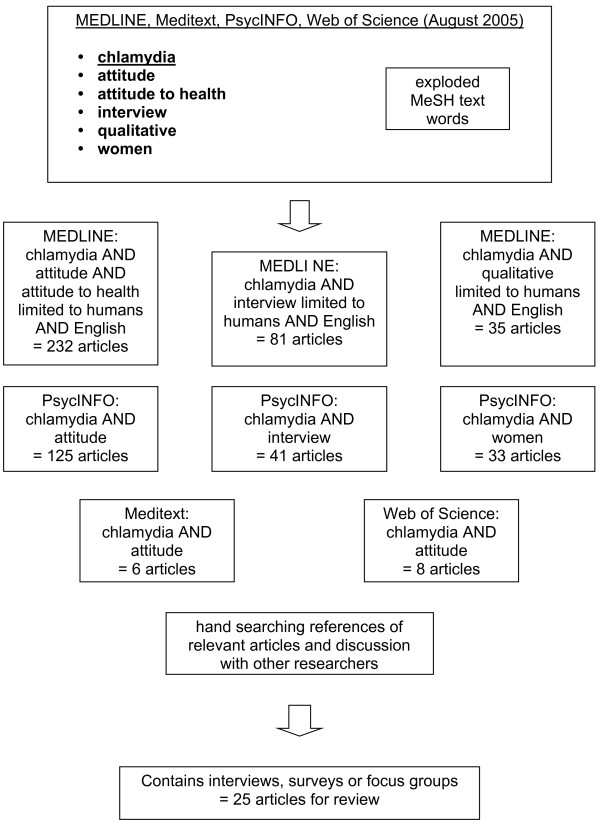
**Literature Search**. Figure 1 shows the search terms used in each database (MEDLINE, Meditext, PsycINFO, Web of Science). A flow diagram illustrates how many articles were generated from each source and the inclusion criteria used to select the 25 articles for full review.

We searched the topic of chlamydia in the databases of MEDLINE, Meditext, PsycINFO and Web of Science up to August 2005. Other search terms used were attitude, attitude to health, interview, qualitative and women. Relevant articles were also identified by hand-searching reference lists of relevant articles and from discussion with Australian researchers in the area. Our database search was repeated by a Melbourne University librarian to validate the list of abstracts found. Abstracts in languages other than English were excluded. The search strategy and inclusion criteria are described in Figure 1.

NP read all 561 English abstracts. Inclusion criteria: about chlamydia, included women, involved interviews/surveys/focus groups, looked at women's views/opinions/attitudes and were published in English. Twenty-five articles were selected for full review. NP read and reread all full text papers retrieved, summarised their settings, subjects and findings, made an assessment of the quality of the different studies and applied the initial thematic analysis. JG, RP, CF and JH were randomly allocated six papers each to read and review to further define and validate NP's summary of the findings, assessment of the quality of the studies and impression of the themes emerging from the data. JG, RP and NP discussed the thematic analysis and reached consensus on the main and recurrent themes which relate to women's views on chlamydia screening, testing and diagnosis. As a group JG, RP and NP compared our thematic analysis with the Theory of Planned Behaviour [11]. We were interested to discover whether the TPB could help draw together our thematic analysis into a useful model for designing a chlamydia screening program. RP has the most expertise with psychological theory within our group and assisted us with the application of the TPB model.

## Results

Of the 561 articles we identified, twenty-five fulfilled the inclusion criteria. Eight of the articles reviewed report on studies that took a predominantly qualitative approach; fourteen on studies that were predominantly quantitative in nature and three on studies that blended qualitative and quantitative approaches. Table 1 sets out those studies including a qualitative component and Table 2 those studies including a quantitative component. Ten studies were from the USA, 12 from the UK and one each from Netherlands, Sweden and Australia respectively. Seven studies were community-based surveys, 18 were based in a clinical setting. The Australian study is an unpublished Master's thesis, the Dutch article and two of the UK articles are published letters. Thematic analysis of the summarised findings from both the qualitative and quantitative studies revealed recurrent themes relating to factors that promote women's acceptance of chlamydia screening and factors that make chlamydia screening less acceptable to women.

### Themes derived from qualitative and quantitative studies

#### What factors promote screening?

There were several common themes identified in our review which relate to factors women think promote acceptance of chlamydia screening and which make a diagnosis of chlamydia easier to deal with.

##### Accurate knowledge about chlamydia [13-21]

These related to the type and level of information women possess about chlamydia and chlamydia screening. Women are more likely to accept screening for chlamydia if they think that chlamydia is a serious condition, if they think that chlamydia is common, if they are aware that a woman who has chlamydia may have no symptoms, if they think that tests are important, if they understand the testing process, if they are aware of the long-term effects of chlamydia infection and in particular if they are aware of the possibility of infertility. One study strongly emphasised the need to focus on the positive aspects of choosing to have chlamydia tests (such as exhibiting responsible self-caring behaviour) rather than focusing on the "negatives" of a chlamydia diagnosis. Women want better access to information about chlamydia via health leaflets, doctors, schools, magazines, billboards and TV ads and some indicated that they feel that humour is an important tool in conveying this information.

##### Feeling chlamydia is personally relevant [13,16,20,22-25]

There were also various personal factors that influenced acceptance of chlamydia screening. These included a woman being more likely accept a chlamydia test if she had a new partner, if she had had "other" non-steady partners in the last six months, if she perceived herself or her partner to be at risk of chlamydia, if she or her partner had symptoms, if she thought she might be pregnant, if she had previously had a diagnosis of chlamydia, if she had a good rapport with the person offering her the test and if she was interested in her own health maintenance.

##### Choice: having multiple options for chlamydia testing [14,18,20,21,26-33]

Another significant theme identified was women's desire for "choice" in how chlamydia screening is offered. Women want to have a range of testing options including urine tests, self-administered swabs, pelvic exams and clinician-collected swabs, home-testing options, self-testing options, outreach health professionals and mobile health vans. Women feel it is important that they are "in control" of chlamydia tests and results. They want to be able to choose to participate in chlamydia screening, to be actively offered screening and to be able to refuse screening.

##### Pragmatic aspects: it needs to be easy [19,20,23,27,34]

Another theme related to pragmatic aspects of chlamydia screening. Women want access to free chlamydia tests. They think incentives for testing would improve uptake. Testing needs to be easy and quick (urine tests have good acceptability). PAP tests are thought to be a good time to offer a chlamydia test.

##### Feeling supported to deal with a chlamydia diagnosis [14,35,36]

Finally themes relating to support needed when dealing with a diagnosis of chlamydia were common. Women want support for partner notification, for dealing with a positive chlamydia diagnosis and with the fear of its future effect on reproductive health. Women feel chlamydia needs to be normalised and destigmatised.

#### What gets in the way of chlamydia screening?

Numerous themes related to factors which make chlamydia screening less acceptable to women

##### Ignorance and inaccurate information about chlamydia [14,18,21,26,35]

Many of these themes also relate to type, level and accuracy of information women possess about chlamydia. Women are less likely to accept chlamydia screening if they have not heard of chlamydia, think chlamydia is a "minor" infection or think chlamydia is uncommon, if they believe chlamydia is very hard to cure, if they think the tests are not accurate and if they think "you would know if you had it" i.e. that there would be symptoms. A woman is also less likely to think that her sex partner (and hence herself) is at risk of chlamydia if her partner is "known" (the definition of "known" varies considerably – may sometimes only be "known" for a few hours).

##### Denial [14,21]

Denial was also identified as a significant theme, including a woman not wanting to acknowledge sexual activity, thinking herself at low risk of having chlamydia, and thinking her partner is at low risk of having chlamydia.

##### Moral connotations and stigma [18,26,35,37,38]

In many studies women reported feeling put off chlamydia screening by the moral connotations of a chlamydia diagnosis. They reported feeling shame, guilt, self-blame, embarrassment, anger, low self-esteem, shock, worry, unhappiness and surprise on being diagnosed with chlamydia. A diagnosis of chlamydia is seen as having a strong stigma attached to it. Some women believe that to use condoms shows distrust of your partner.

##### Fear and anxiety [14,26,36]

Themes of fear and anxiety were common. Women reported feeling fearful about infertility and future reproductive health, anxious about partner notification and worried about the negative effect of a chlamydia diagnosis on their personal relationships.

##### Confidentiality and privacy concerns [13,14,22,23]

Many women had confidentiality and privacy concerns. These included concerns about the confidentiality of attending a clinic and of results, wanting to keep STI screening private, not wanting anyone to know and thinking General Practice is not confidential or private enough for chlamydia testing.

##### Pragmatic aspects – women are put off if screening is difficult or uncomfortable [13,18,23,32]

Pragmatic aspects were again important. Women found the time and cost involved in a having a chlamydia test put them off being tested. Having to physically go to a clinic as well as discomfort with sample collection (especially pelvic examinations and physician-collected swabs) were also barriers to accepting chlamydia screening.

### Synthesising the findings using the Theory of Planned Behaviour

**Figure 2:**
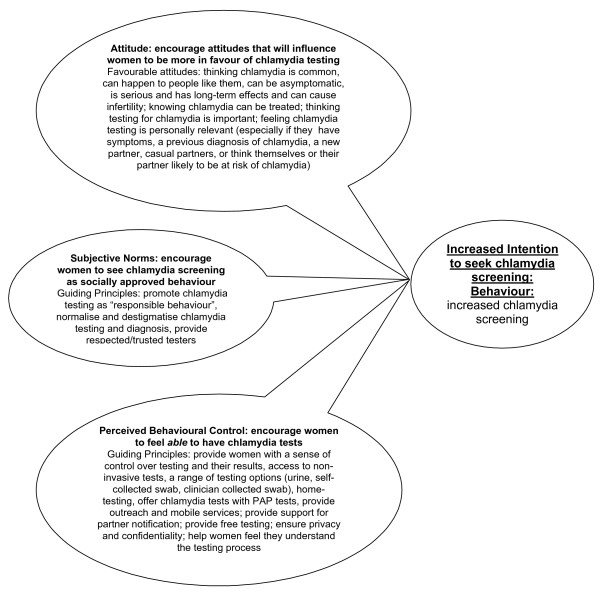
**Application of the Theory of Planned Behaviour**. Figure 2 uses Ajzen's Theory of Planned Behaviour to generate a conceptual model to aid in understanding how the various themes identified influence women's acceptance or rejection of chlamydia screening

The Theory of Planned Behaviour (TPB) [11] helps to understand the factors that affect the human decision-making process. The themes derived from our review appear to accord well with the concepts of Attitude, Subjective Norms and Perceived Behavioural Control inherent in the TPB. Figure 2 illustrates how the TPB can be used to group findings from the thematic analysis in a way that could usefully inform the development of a public health campaign or chlamydia screening program. Some of the data in our themes did not fit into the TPB model, notably women's desire for better access to information on chlamydia, the suggestion regarding the use of humour to promote chlamydia awareness, and finally women's desire to be offered screening but to feel able to refuse. The area of Subjective Norms was less supported by our review than either Attitude or Perceived Behavioural Control but still has adequate data. Overall the TPB appears to be a useful way of conceptualising the findings of this literature review and may assist in chlamydia screening program planning and implementation.

## Discussion

The studies reviewed report on the views of a total of 26 256 women from five countries (98% from the UK and USA) on chlamydia screening, testing and diagnosis. Major themes identified relate to the need for knowledge and information, choice and support and to concerns about confidentiality, cost and stigma. Fear and anxiety were commonly expressed.

### Major findings

While it is clear that accurate knowledge about chlamydia is fundamental to women's increased acceptance of screening, the literature suggests that numerous factors impinge on women's incorporation and potential application of such knowledge. Some of these factors are more amenable than others to public health measures. Denial, for instance, refers to a self-protective, defensive mode of thinking which is adopted in the face of rational evidence [39]. Personal moral views may also be maintained impervious to any presentation of relevant facts. Nevertheless the challenge remains to devise creative approaches to the way knowledge is presented which not only inform but simultaneously normalise and destigmatise the subject-matter, allowing it to become personally relevant to those whose tendency may have been to shrug it off. The literature surveyed contained at least one relevant pointer when respondents singled out for mention the importance of humour. The belief that to wear a condom shows distrust of your partner is obviously one which bypasses the rational and maintains a hold which is not easily loosened. Such challenges suggest the importance of taking a broad, multi-disciplinary approach to devising public health programs. Somewhat less difficult to address, although still personal in nature, are the factors grouped under the subheading of fear and anxiety, particularly if opportunities are built into programs for discussion of fears and for sensitive, tangible support regarding partner notification. Regarding women's reported concerns about privacy and confidentiality, we need to consider whether present safe-guards are, in practice, adequate and be ready to identify and implement any necessary improvements. We need to be sensitive to fears in this area and develop systems and visible modes of operation which go some way to allaying such fears of the public. Feedback on the pragmatic aspects of testing contains important detail giving us positive direction about what women find important. In the light of Ajzen's TPB it is important to ensure that programs have elements aimed at addressing Attitude, Subjective Norms and Perceived Behavioural Control. Attitude: encourage attitudes that will influence women to be more in favour of chlamydia testing. Subjective Norms: encourage women to see chlamydia screening as socially approved behaviour. Perceived Behavioural Control: encourage women to feel *able *to have chlamydia tests.

### Limitations

Our review is limited in that we only included studies written in English and also by the fact that 98% of the studies selected deal with the views of women from the USA and UK. The quality of the studies reviewed also varied considerably. Some of the qualitative studies had very small number of participants and participant selection was often non-random [18,35]. For example one study which used a qualitative approach reports on a small cross-sectional study interviewing only four women with positive chlamydia tests [38]. The scope of the research questions addressed by some of the quantitative studies was sometimes limited to "is urine testing acceptable to women?"[28,34] or even just "do women return a urine chlamydia test kit?"[33]. However despite this variability all studies had similar findings. It is striking that the findings support each other strongly, irrespective of whether the studies were conducted using predominantly qualitative or quantitative approaches.

In our thematic analysis we relied initially on the work of NP, with the rest of the team discussing her suggested themes subsequently. This may have biased our thematic analysis although it is hoped that our team discussion helped to validate the theme definitions. A further limitation of our study relates to the timing of our use of the TPB. We applied the TPB sequentially following our thematic analysis. This may limit the applicability of the TPB model we have derived, as some important information may have been missed. Nevertheless it proved a useful tool to us as we processed our findings. The research team vigorously discussed the suggested themes and a subgroup worked on applying the TPB. We reached consensus on our themes and our application of the TPB model.

### Recommendations

From our review we can draw some lessons for designing an effective chlamydia screening program aimed at women. It is important to raise awareness about chlamydia in the community to be screened, to increase the access of young women in particular to accurate information about chlamydia and to explore creative ways to normalise and destigmatise chlamydia. Chlamydia testing needs to be free, easy to access, private, as non-invasive as possible and should be available in a variety of settings. Beyond facilitating the actual diagnosis, support emerges as equally crucial, to help women cope with a diagnosis of chlamydia and to assist them with partner notification.

Although these generalised findings emerged from studies involving multi-ethnic groups from more than one country, it may be important in the Australian context for specific studies to explore the views of various groups of young Australian women, including those of Aboriginal women in urban, rural and remote areas.

## Conclusion

Our review looks at the views on chlamydia testing, screening and diagnosis of more than 26 000 women from 5 countries (predominantly the UK and USA). Women from various countries and ethnic backgrounds shared similar views. These are significant considerations when planning future chlamydia screening programs and public health initiatives. Our review suggests women will be more in favour of chlamydia testing and screening if they think chlamydia is common, can happen to people like them, can be asymptomatic, is serious and has long-term effects, can cause infertility, if they know chlamydia can be treated and if they think testing for chlamydia is important. It may be helpful to promote chlamydia testing as "responsible behaviour," to normalise and destigmatise chlamydia testing and diagnosis and to offer testing by respected/trusted testers. Women may be more likely to accept chlamydia screening if they have access to non-invasive tests, to a range of testing options (urine, self-collected swab, clinician-collected swab); home-testing, outreach and mobile services, if chlamydia testing is free, if they are provided with support for partner notification and if they feel confident that their privacy and confidentiality will be maintained. While these criteria hold true across national and ethnic differences in the populations which were the subject of the literature reviewed to date, the possibility arises that other groups may have a unique point of view. In terms of developing a chlamydia screening program for Australia this highlights the need for specific further research on Australian women's views about chlamydia screening. testing and diagnosis, before any one chlamydia screening program is implemented.

## Competing interests

The author(s) declare they have no competing interests.

## Authors' contributions

NP carried out the literature search, summarised the articles found and carried out the thematic analysis. JG participated in the design of the search strategy and in the coordination of the overall project. NP, JG, RP, JH and CF were all involved in reviewing the articles retrieved. All authors read and approved the final manuscript.

## Pre-publication history

The pre-publication history for this paper can be accessed here:



## Figures and Tables

**Table 1:** Summary of the studies that included a qualitative component. Describes the settings, subjects and findings of the 11 studies that included a qualitative component.

	**Setting**	**Subjects**	**Study**	**Findings**
[14]	USA, non-clinical and non-medical setting	32 men (aged 15–20 years) and 23 women (aged 16–24 years). Participants were sourced half from a job program and half from the juvenile justice system. All were out of school and were deemed "high risk" for STI acquisition.	Qualitative: 8 focus group interviews	Barriers to screening: confidentiality and privacy concerns; fear of a positive diagnosis and the negative consequences of chlamydia infection; ignorance of the significance of a chlamydia diagnosis Facilitators of screening: the option of home testing, improved information about chlamydia; support for dealing with a diagnosis of chlamydia; promotion of chlamydia screening as "exhibiting responsible behaviour"

[35]	Sweden, urban, youth clinic	5 women and 4 men aged 18–22 years. All had a positive chlamydia test and were patients of the interviewer. All the men were contacted through partner notification.	Qualitative: In depth interviews	Attitude to being diagnosed with chlamydia: shame/guilt (worse for women); satisfaction (more for men). Support: Women told mothers, sisters and friends; men told no one. Beliefs: having sex with someone "known" protects against catching an STI; using condoms with someone you like is an expression of distrust; men felt women should be "condom-promoters"

[15]	UK, urban, sexual health clinic (London)	12 men (16–44), 12 women (16–34), heterosexual and a chlamydia diagnosis (82% participation)	Qualitative: Semi structured interviews	Attitude to chlamydia diagnosis: worry about infertility; self-blame Knowledge: some people thought chlamydia was a relatively minor infection and put off seeking treatment

[23]	UK, urban, 2 genitourinary medicine (GUM) clinics and 1 family planning (FP) clinic (East Midlands)	37 women (15–53) had been screened for chlamydia, 3=black, 34=white, socially diverse backgrounds. All women who agreed to participate were included.	Qualitative: Semi structured interviews	Reasons for STI screening: own symptoms; partner's symptoms; partner's behaviour; own behaviour and health maintenance Barriers to STI screening in General Practice: relationship with the GP; views of the quality of clinical care; need for confidentiality and accessibility Facilitators to STI screening: raise concerns about STIs at the same time as Pap tests; help women feel "in control" of receiving their test results

[36]	UK, urban, genitourinary medicine (GUM) clinic, family planning (FP) clinic (Glasgow)	17 women (18–29) with a current or recent diagnosis of chlamydia. The first 17 who agreed to participate were included (10 from GUM and 7 from FP response rate = 62%)	Qualitative: Semi structured interviews	Attitude to chlamydia diagnosis: stigma attached to having an STI; stigma about attending a sexual health service; anxiety about partner notification; fears about future effects on reproductive health and fertility. Facilitators: normalise and destigmatise chlamydia diagnosis; provide support for partner notification; provide support for dealing with uncertainties regarding future infertility

[26]	USA; population based non-medical setting	120 men and women aged 18–25, 55% were female (n = 66) and 61% were aged 18–21, 41% were white, 22% black and 33% Latino.	Qualitative: Semi structured Computer-Assisted Telephone Interviews (CATI)	Attitude to chlamydia diagnosis: shock, anger, embarrassment, disappointment (50% of participants); relief (14%); negative effect on personal relationships; fear; depression; low self-esteem; health-related concerns (only 4 participants); would seek treatment for chlamydia (most participants). Knowledge: 25% of participants believed chlamydia is very difficult to treat. Barriers to chlamydia testing: cost/time involved in seeking treatment; stigma. Facilitators of chlamydia testing: home urine testing/urine self-test kits. Advantages to home testing: privacy, increased testing, convenience, lower cost (no need to see a doctor). Disadvantages to home testing: doubts about test accuracy, doubt whether people at risk would use home tests and whether people with a positive result would get treated. Advantages of being given results over the phone: privacy, convenience, learning STD status, having control over the process Disadvantages of being given results over the phone: risk of a positive test and no professional to talk to face-to-face, fear of a positive test or of being overheard might put people off calling.

[18]	Australia, urban, youth clinic (Melbourne)	25 homeless young people aged 16–26; 19 male and 6 female, level of schooling varied from Year 8 to Year 12	Qualitative: 6 focus groups with 4 to 6 participants.	Participants had limited knowledge about chlamydia. Suggested ways to disseminate information: school, free entertainment magazines, billboard advertising, TV ads and pamphlets distributed by community groups. Humour seen as a useful tool. Barriers to screening: embarrassment, cost, ignorance. Suggested facilitators: mobile health van, free medical care (bulk billing), improved advertising and awareness of chlamydia, incentives for testing, outreach health professionals, self-testing.

[21]	UK, urban, general practice (8 general practices) (Edinburgh)	20 women who had participated in a pilot of opportunistic chlamydia screening including both those with positive (4) and negative (14) results and those still waiting for results (2).	Qualitative: Semi-structured interviews	Perception of risk of chlamydia infection: most women said they were not worried whilst waiting for results as they did not perceive themselves as being "at risk". Most had not heard of chlamydia before and therefore thought it was uncommon. Women found it difficult to accept that chlamydia could be asymptomatic. Women based their assessment of the risk of chlamydia infection on its perceived prevalence, on their previous experience, on their own or their partner's sexual history and on the presence or absence of symptoms. Views about chlamydia screening: all women were glad to have been screened. Reasons included: awareness of the risk of infertility, the ease of testing and knowing it can be treated with antibiotics. Information: This was important both in deciding whether to accept a test and in dealing with results. Knowing that if they did have chlamydia it could be treated with antibiotics was important to several women. Knowing that chlamydia could be asymptomatic for some time was important for women in dealing with the implications of an infection. Choice: Many women felt that although routine screening was a good idea, choosing to participate or not was very important. Younger women however thought being offered screening directly was good as they might not have asked for it.

[34]	UK, urban, population based probability sample (London)	36 sexually experienced men and women aged 18–44 (equal numbers of men and women)	Qualitative: In depth face-to-face interviews	Factors influencing an individual's decision to participate in chlamydia screening by urine included: trust and rapport with the interviewer who was inviting them to have the test, understanding the aims of the test and what would happen to the urine sample, a sense of obligation, not feeling embarrassed, the perceived importance of the test and the opportunity to receive free testing.

[38]	UK, urban, family planning clinic (Nottingham-shire)	4 women aged <25 who had had a positive chlamydia result 6 months previously.	Qualitative: In depth face-to-face interviews	Feelings about a diagnosis of chlamydia included: shock, worry, unhappiness, embarrassment and surprise. Attitude to partner notification: 3 felt embarrassed about the need to trace contacts but all 4 women attempted to do so. Concern for the future: all were very concerned about the long term effects of chlamydia if left untreated.

[20]	UK, urban, general practice, family planning (FP), genitourinary medicine (GUM) clinics, adolescent sexual health clinics, termination of pregnancy clinics and women's services in hospitals (antenatal, colposcopy, gynaecology and infertility clinics).	25 sexually active women aged 16–24 attending a range of healthcare settings, who had been screened for chlamydia, completed a questionnaire on the acceptability of screening and volunteered to participate in an in depth interview on their views on chlamydia screening. (80 people gave their contact details but only 25 were available for interview)	Qualitative: In-depth interviews	Women were pleased to be offered screening. A minority were concerned that the offer of screening meant they had been singled out as being "at risk" of an STI. Participants attending GUM or FP clinics were most comfortable with being screened. Motivations for accepting screening included: awareness that chlamydia infection can be asymptomatic, (many reported that they would not have sought out screening if it had not been opportunistically offered to them), also exposure to "formal" information (health professionals, leaflets, publicity campaigns) and "informal" information (family and friends). Women felt able to refuse screening. The most important factors influencing the decision to accept screening were having the perception of being at risk of infection, awareness of the possible long-term effect on fertility and thinking that chlamydia was easily treated. All the women interviewed found giving a urine sample acceptable. The majority said they would have declined screening if it involved a clinician-collected swab.

**Table 2:** Summary of the issues addressed in the studies that included a quantitative component. Summarises the research questions addressed in the studies that included a quantitative component, the number of studies asking each question, the location of the studies and number of women represented in combined studies from each location, as well as the median and range of responses to each research question.

**Research question**	**Number of studies asking question**	**Location of studies and number of women in combined studies from each location**	**Median and range of responses (median calculated by placing the response percentages in value order and finding the middle value)**	**Ref**
Number of women accepting urine screening for chlamydia	4	UK (n = 27470)	71–83% (median 76%) of women accepted screening for chlamydia by urine	[20, 25, 28, 34]

Readiness to seek chlamydia screening on partner change	3	USA (n = 736)	28–57% (median 47%) of women reported they currently seek chlamydia screening with partner change; 28–50% reported they are ready to screen on partner change; 11–15% are thinking about being ready to seek screening on partner change and 4–7% are not ready to seek screening on partner change	[13, 16, 22]

Knowledge of chlamydia	3	UK (n = 737)	60–93% of women had heard of chlamydia, 26–86% knew chlamydia could be asymptomatic and 68–82% were not aware of the consequences of untreated infection.	[17, 19, 38]

Acceptability of chlamydia screening in different healthcare settings: general practice, family planning clinics, termination of pregnancy clinics, colposcopy clinics, antenatal clinics, youth clinics, infertility clinics, genitourinary medicine clinics, gynaecology clinics	2	UK (n = 22126)	Acceptability of chlamydia screening in these healthcare settings ranged from 38–100% (median 82%)	[19, 20]

Acceptability of self administered swab (SAS) vs. first void urine (FVU) vs. pelvic exam and clinician-collected swab for chlamydia screening	3	USA (n = 2093)	All 3 studies showed FVU was the preferred method of chlamydia screening over SAS and pelvic examination but FVU and SAS were similar in ease and acceptability.	[27, 29, 30]

Participation in home screening for chlamydia and preferred test (urine or vaginal swab)	3	UK (n = 208), USA (n = 98), Holland (n = 189)	Participation rate in home testing ranged from 31–52% (median 39%). Urine testing was more often accepted than vaginal swab testing: 47–63% relative to 32–37%.	[31–33]

Rates of partner notification following a diagnosis of chlamydia	2	UK (n = 73), USA (n = 55)	Both studies report a 75% rate of partner notification	[24, 25]
